# From entertainment to persuasion: how attitudes toward advertising shape responses to brand placement and television advertising

**DOI:** 10.3389/fpsyg.2026.1963379

**Published:** 2026-10-05

**Authors:** Linshang Li

**Affiliations:** School of Foreign Languages, Guangzhou Nanfang University, Guangzhou, Guangdong, China

**Keywords:** attitude toward advertising, brand attitude, brand placement, consumer decision-making, media psychology, purchase intention, television advertising

## Abstract

**Introduction:**

Embedded brand communication is increasingly encountered alongside explicit advertising, yet audience-level differences may determine which format is more persuasive.

**Methods:**

In a randomized online experiment, adults recruited from a United States consumer panel were assigned across seven cells representing brand placement, 30-s television advertising, combined exposure, or control for Heinz and Snapple. Participants viewed full-length sitcom content and completed multi-item measures of attitude toward advertising (AttADV), brand attitude, and purchase intention.

**Results:**

General attitudes toward brand placement were more favorable than attitudes toward conventional television advertising. Exposure format interacted with AttADV for both focal brands: at low AttADV, placement produced more favorable brand attitudes than a 30-s advertisement, whereas no statistically significant difference between formats was detected at mean or high AttADV; the study was not designed to establish equivalence at these levels. Combined exposure produced no statistically significant improvement in brand attitude or purchase intention relative to either single-exposure condition, though the study was insufficiently powered to rule out small incremental effects.

**Discussion:**

The findings support an audience-by-format account of persuasion in which embedded brand communication is most advantageous among viewers who are less receptive to conventional advertising. They also indicate that additional cross-format exposure does not necessarily yield incremental benefit. Testing the same psychological pattern across two familiar brands within full-program viewing provides a comparatively naturalistic basis for examining persuasion in increasingly integrated media environments.

## 1 Introduction

Commercial persuasion is increasingly woven into the media experiences that audiences choose for information and entertainment. Viewers may encounter a brand during a television narrative, in a sponsored social-media post, inside a streaming interface, or in a conventional advertising break. These formats differ in how clearly; they interrupt the surrounding content and how readily they signal persuasive intent (Bui, 2026; Li et al., 2026). From a media-psychology perspective, this distinction is important because responses to advertising are shaped not only by message content but also by the attentional, affective, and interpretive context in which the message appears. Recent work shows that television context can carry over to subsequent advertising responses, while research on native and feed-based advertising indicates that integration with surrounding content can alter attention and evaluations (Yao and List, 2026).

Brand placement is a prominent form of embedded commercial communication. Instead of appearing as a discrete advertising interruption, the brand becomes part of the visual or narrative environment of the program. Brand placement effects are therefore inseparable from the way audiences process characters, plot events, and program-related information. Russell (2002) showed that modality and plot connection jointly influence memory and attitude toward placed brands, subsequently emphasized that placement outcomes depend on executional features, processing conditions, and audience characteristics (Fan et al., 2025). These findings imply that the persuasive value of placement cannot be reduced to exposure alone; it depends on how the brand is encountered within the media experience.

One mechanism that helps explain differences between embedded and explicit advertising is persuasion knowledge. The Persuasion Knowledge Model proposes that people learn to recognize marketers’ goals and tactics and use that knowledge to interpret and manage persuasion attempts. A conventional television commercial announces its commercial purpose by its format and position within an advertising break. A placement can make that purpose less focal because the brand is processed alongside narrative content (Vargas-Salas et al., 2026). This does not mean that placements are inherently covert or immune to resistance: prominent, incongruent, or clearly disclosed placements may also activate persuasion knowledge. The central issue is that embedded and interruptive formats create different conditions for recognizing and responding to persuasive intent.

Evidence from newer media environments reinforces the importance of contextual integration. The advertising was associated with more favorable advertising and brand attitudes than traditional display advertising, and showed that contextual mobile advertising characteristics can influence purchase intention through advertising attitudes. At the same time, eye-tracking evidence indicates that even subtle advertising cues in native formats can trigger rapid disengagement once users recognize the post as commercial (Szabó and Mitev, 2026). Together, these findings point to a general principle: the psychological effect of an advertising message is partly determined by how it fits into the surrounding media environment and by whether audiences interpret that encounter as intrusive persuasion.

Audience predispositions may be equally important. Attitude toward advertising in general (AttADV) refers to a relatively broad evaluative orientation toward advertising rather than an evaluation of one particular advertisement. Consumers vary in the extent to which they perceive advertising as informative, entertaining, irritating, manipulative, or socially useful. Pollay and Mittal (1993) linked general advertising attitudes to beliefs about advertising’s personal and social consequences, while Mehta (2000) showed that favorable attitudes toward advertising are associated with stronger advertising responses (Jayapal and Harishree, 2025). AttADV can therefore function as a psychological lens through which later persuasive encounters are interpreted. This is consistent with broader evidence that media consumption actively interacts with pre-existing sociocultural orientations and identity-based heuristics to systematically reshape audience evaluations and attitudes (Liu et al., 2026; Taylor et al., 2023).

This individual difference is especially relevant when brand placement is compared with a conventional commercial. Viewers who already respond favorably to advertising may have little reason to penalize an explicit 30-s advertisement, reducing any relative advantage of an embedded format. By contrast, viewers who are unfavorable toward advertising may be more likely to ignore, discount, or resist an overt commercial. For these viewers, a brand encountered within entertainment content may be evaluated under less defensive processing conditions (Milovan et al., 2025). The resulting prediction is conditional rather than absolute: placement should be most advantageous when the audience is relatively unreceptive to advertising, and the difference between placement and conventional advertising should narrow as AttADV becomes more favorable.

The present study focuses on two downstream responses: brand attitude and purchase intention. Brand attitude captures an overall evaluation of the focal brand, whereas purchase intention reflects the reported likelihood of considering or seeking the product. The constructs are related but not interchangeable. A persuasive exposure can alter evaluation without producing an equivalent change in purchase intention because purchase decisions are also constrained by need, price, availability, prior experience, and category involvement (Orús et al., 2026). Treating the two outcomes separately therefore provides a more informative account of how media exposure may translate into consumer decision processes.

Three limitations in the existing literature motivate the present design. First, a large share of placement research has relied on short clips or isolated advertising stimuli, which provide experimental control but reduce the continuity of normal program viewing (Deryl et al., 2023). Second, studies frequently emphasize average differences between persuasive formats while paying less attention to stable audience orientations that can determine for whom those differences matter. Third, single-format comparisons do not address the increasingly common situation in which the same brand appears both within content and in an explicit advertisement during the same viewing experience (Rodriguez and Sangle-Ferriere, 2023). These issues make it useful to compare placement-only, advertising-only, combined-exposure, and control conditions while simultaneously examining an audience-level moderator.

Accordingly, this study compares brand placement with a conventional 30-s television advertisement using full-length sitcom content featuring two established consumer brands, Heinz and Snapple. AttADV is treated as a boundary condition for the placement-versus-advertising contrast in brand attitude, while separate analyses assess purchase intention and whether combined exposure provides an incremental benefit over either single format (Lu et al., 2026). The analytic framework is summarized in Figure 1. By integrating media context with advertising receptivity and by examining the pattern across two brands in a full-program setting, the study aims to clarify when embedded persuasion is most effective and when additional exposure fails to improve consumer response.

**Figure 1:**
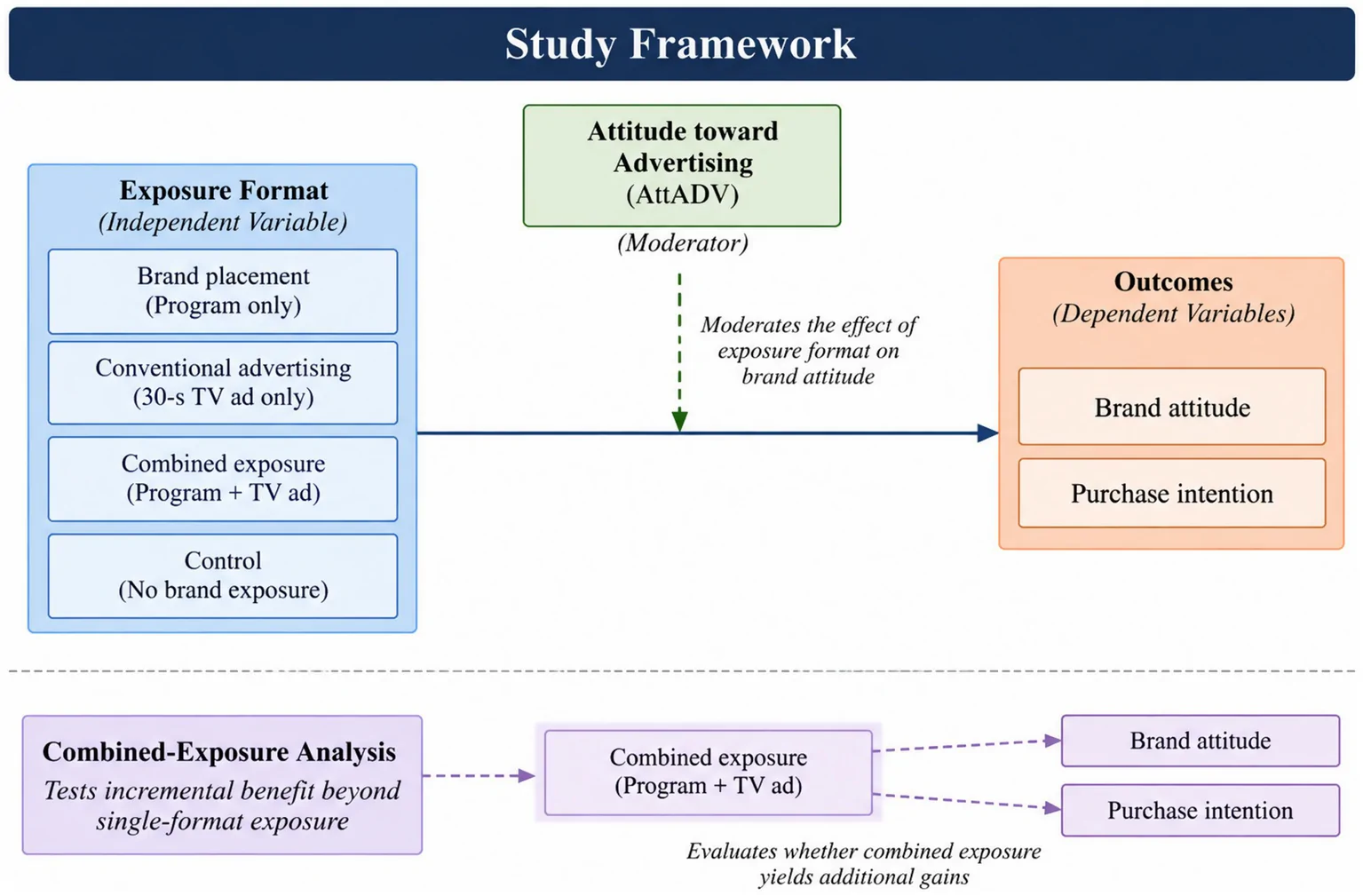
Study framework. Exposure format is evaluated in relation to brand attitude and purchase intention. Attitude toward advertising (AttADV) is modeled as a moderator of the placement-versus-advertising contrast for brand attitude, and the combined-exposure analysis tests incremental benefit beyond single-format exposure.

## 2 Materials and methods

### 2.1 Participants and research design

A randomized online experiment was conducted with adults recruited from a large United States consumer research panel. A total of 775 individuals were initially recruited, of whom 527 completed the study within the specified data-collection window (completion rate: 68%). Cell sizes in the final analytic sample ranged from 42 to 83 per condition; full cell-level sample sizes are reported in Table 1. A total of 248 participants did not complete the study. Because demographic information was not available for all non-completers, formal attrition analyses comparing non-completers with the final analytic sample on age, gender, and household income could not be conducted. Differential attrition therefore cannot be ruled out and should be considered a limitation of the study. Participants represented a range of educational and household-income backgrounds. The experiment used a between-subjects design with seven randomized cells. For each of the two focal brands, Heinz and Snapple, participants received one of three active exposure conditions: brand placement only, a conventional 30-s television advertisement only, or combined exposure to both formats.

**Table 1:** Brand attitudes and purchase intentions by exposure condition.

Outcome	Brand placement M (SD) [*n* = 79]	Advertising M (SD) [*n* = 79]	Placement + advertising M (SD) [*n* = 78]	Control M (SD) [*n* = 42]	*F*	*p*	η^2^	**Power (**f = 0.10)
Heinz: brand attitude	6.22 (0.80)	5.84 (1.15)	5.69 (1.08)	5.18 (1.25)	1.95	0.144	0.016	0.257
Heinz: purchase intention	5.80 (1.19)	5.72 (1.27)	5.69 (1.44)	4.85 (1.72)	1.04	0.355	0.009	0.248
Snapple: brand attitude	5.43 (1.04)	5.12 (1.37)	5.22 (1.46)	4.74 (1.15)	0.54	0.583	0.004	0.270
Snapple: purchase intention	4.74 (1.67)	4.74 (1.78)	4.43 (1.81)	3.77 (1.85)	0.49	0.613	0.004	0.260

*n* values reflect the number of participants per condition. Heinz conditions: brand placement *n* = 79, advertising *n* = 79, combined *n* = 78, control *n* = 42. Snapple conditions: brand placement *n* = 83, advertising *n* = 83, combined *n* = 83, control *n* = 42. Minor variation in purchase-intention degrees of freedom reflects listwise exclusion of missing responses on that measure.

A seventh cell served as the control and contained neither focal-brand placement nor focal-brand advertising. Conceptually, the design therefore compared four exposure categories - placement, advertising, combined exposure, and control while testing the active conditions separately for each brand. After completing demographic data, participants were instructed to watch the assigned program in its entirety. The focal purpose of the experiment was not disclosed before exposure. Immediately after viewing, participants completed measures of general attitude toward television advertising, attitude toward brand placement, brand attitude, and purchase intention.

### 2.2 Stimulus development

The stimulus materials were constructed from two episodes of the sitcom Two and a Half Men that contained clearly identifiable appearances of Heinz and Snapple. The focal sequences were selected because the brands were visible, used by central characters, and integrated into the ongoing action rather than appearing as isolated background props. In the Heinz sequence, the product appeared on a table during an interaction between principal characters and was subsequently handled within the scene. In the Snapple sequence, a bottle appeared in the narrative environment and was visibly handled and consumed by a principal character. Both placements therefore combined visual prominence with character-product interaction.

Three versions of the program were prepared for each focal brand. Participants in the placement condition viewed the program containing the focal placement without a conventional advertisement for that brand. Participants in the advertising condition viewed a 30-s focal-brand commercial during a commercial break without the corresponding placement. Participants in the combined condition encountered both the placement and the 30-s advertisement during the same viewing session. The control version contained neither focal-brand exposure. Commercial breaks contained advertisements for unrelated established brands so that the target commercial appeared within a broader advertising sequence rather than as an isolated laboratory stimulus. The use of full-length program content was intended to preserve the narrative, character, and attentional context in which embedded and interruptive advertising are ordinarily processed. Within the advertising condition, the focal brand commercial was placed in the second position of the commercial break to standardize its serial position relative to the filler advertisements; this positioning was held constant across all advertising and combined-exposure conditions.

### 2.3 Stimulus validation: expert review

Before the main experiment, eight academics and advertising professionals participated in an expert review of the stimulus materials. They assessed focal-brand visibility, character interaction with the product, and the degree of narrative integration for both Heinz and Snapple. Both placements were judged to be clearly identifiable, to involve meaningful interaction with principal characters, and to demonstrate moderate-to-high integration with the program narrative. The filler advertisements were also reviewed by the same panel, and no substantive concerns were identified. No separate participant-level brand-recognition manipulation check was conducted; this limitation is acknowledged in Section 4.7.

### 2.4 Measures

#### 2.4.1 Attitude toward advertising

Attitude toward advertising was operationalized as participants’ general evaluation of conventional television advertising. Three five-point items adapted from Donthu and Gilliland (1996) assessed perceived usefulness and the tendency to attend to or disregard television advertising. Item scores were averaged so that higher values indicated a more favorable orientation toward advertising. The scale showed acceptable internal consistency (M = 3.16, SD = 0.89, Cronbach’s alpha = 0.77).

#### 2.4.2 Attitude toward brand placement (AttBP)

A parallel three-item, five-point measure assessed participants’ general orientation toward brands incorporated within television programming. The item wording mirrored the advertising-attitude measure while referring to embedded brand appearances. Higher scores represented more favorable attitudes toward brand placement. Internal consistency was acceptable (M = 3.49, SD = 0.68, alpha = 0.78).

#### 2.4.3 Focal brand attitude

Focal brand attitude was measured with a six-item, seven-point semantic-differential scale adapted from Maheswaran and Sternthal (1990). Items captured overall favorability and comparative evaluation of the focal brand. Responses were averaged separately for Heinz and Snapple, with higher values indicating a more favorable brand evaluation. Reliability was satisfactory for Heinz (M = 5.85, SD = 1.13, alpha = 0.83) and Snapple (M = 5.12, SD = 1.36, alpha = 0.81).

#### 2.4.4 Purchase intention

Purchase intention was assessed with three seven-point items adapted from Baker and Churchill (1977). The items measured willingness to purchase the focal product if encountered in a retail setting and the likelihood of deliberately seeking the product. Responses were averaged to form a purchase-intention score for each brand. Reliability was acceptable for Heinz (M = 5.67, SD = 1.41, alpha = 0.82) and high for Snapple (M = 4.56, SD = 1.75, alpha = 0.95). Descriptive statistics and reliability coefficients for all measures are reported in Table 2.

**Table 2:** Descriptive statistics and internal consistency of the study measures.

Measure	M	SD	Cronbach alpha
Attitude toward advertising	3.16	0.89	0.77
Attitude toward brand placement	3.49	0.68	0.78
Brand attitude: Heinz	5.85	1.13	0.83
Brand attitude: Snapple	5.12	1.36	0.81
Purchase intention: Heinz	5.67	1.41	0.82
Purchase intention: Snapple	4.56	1.75	0.95

### 2.5 Statistical analysis

Attitude toward advertising (AttADV) and attitude toward brand placement (AttBP) were each measured on five-point response scales, whereas brand attitude and purchase intention were assessed on seven-point scales. Because these constructs entered the regression models as conceptually distinct predictors and outcomes rather than as components of any combined composite, no scale standardization or transformation was performed; the different response formats do not affect the validity of the regression coefficients, interaction terms, or ANOVA comparisons. Prior to the main analyses, manipulation check data were examined to confirm that placement stimuli successfully activated brand awareness in the placement and combined-exposure conditions. Analyses were conducted in three stages. First, a paired-samples t-test compared participants’ general attitudes toward brand placement and conventional television advertising. Second, hierarchical moderated-regression models tested whether AttADV altered the relationship between exposure format and brand attitude. The focal contrast compared placement with 30-s advertising. Exposure format and mean-centered AttADV were entered as predictors, followed by their interaction.

When the interaction was significant, conditional effects were examined at one standard deviation below the AttADV mean, at the mean, and at one standard deviation above the mean. These conditional analyses were implemented with the PROCESS approach to moderation analysis. To address the possibility that AttADV functioned as a proxy for general placement favorability rather than as a distinct dispositional moderator, attitude toward brand placement (AttBP) was entered as an additional covariate in a supplementary set of moderation models; results of this covariate-adjusted analysis are reported in Section 3.3. Third, one-way analyses of variance (ANOVAs) constituting Stage 3 of the analysis compared the placement-only, advertising-only, and combined-exposure conditions for brand attitude and purchase intention, separately for Heinz and Snapple. These tests assessed whether exposure to both persuasive formats produced an incremental advantage over either format alone. All significance tests were two-tailed with alpha = 0.05.

### 2.6 Ethical considerations

The study received ethical approval from the Academic/Ethics Committee of Guangzhou Nanfang University (Reference No. GNFU-SS-REC-2025-187) on 27 December 2025. The committee reviewed the proposed research, “From Entertainment to Persuasion: How Attitudes Toward Advertising Shape Responses to Brand Placement and Television Advertising,” with regard to potential scientific and ethical issues. The committee determined that the study complied with the relevant ethical requirements for research involving human participants and conformed to the principles of scientific ethics. The approval further specified that participation should be voluntary, informed consent should be obtained, participants’ privacy and confidentiality should be protected, and collected data should be used solely for legitimate academic research purposes. The study was assessed as presenting no more than minimal risk to participants. The study was conducted in full accordance with the approved protocol; participant recruitment, consent procedures, and data handling are described in Section 2.1.

## 3 Results

### 3.1 General attitudes toward brand placement (AttBP) and television advertising (AttADV)

Participants evaluated brand placement more favorably than conventional television advertising. Mean attitude toward brand placement was 3.49 (SD = 0.68), compared with 3.16 (SD = 0.89) for AttADV. The paired comparison was statistically significant, *t*(400) = 11.67, *p* < 0.001. The result indicates that embedded and interruptive commercial communication were not evaluated equivalently at the general-attitude level.

### 3.2 Exposure effects relative to control

The active exposure conditions generally produced more favorable brand responses than the unexposed control condition. For Heinz, placement exposure differed significantly from control, *t*(167) = 4.97, *p* < 0.001. The corresponding comparison for Snapple was also significant, *t*(173) = 2.15, *p* = 0.033 (Deryl et al., 2023). Conventional advertising likewise produced a significant difference from control for Heinz, *t*(179) = 4.08, *p* < 0.001, and a borderline difference for Snapple, *t*(179) = 1.97, *p* = 0.050. These comparisons provide evidence that the focal placement and advertising stimuli were capable of shifting post-exposure evaluations.

### 3.3 Moderating role of attitude toward advertising

The central analysis tested whether the relative effect of placement versus conventional advertising varied with AttADV, with a supplementary analysis entering attitude toward brand placement (AttBP) as a covariate to isolate the moderating contribution of AttADV from participants’ general disposition toward embedded formats. A significant exposure-format by AttADV interaction emerged for both focal brands. For Heinz, adding the interaction improved the model, Delta *F*(1,143) = 4.73, *p* = 0.031, and the interaction coefficient was negative, *b* = −0.16, *t*(143) = −2.20, *p* = 0.029. A comparable pattern emerged for Snapple, Delta *F*(1,149) = 5.75, *p* = 0.018, *b* = −0.17, *t* = −2.40, *p* = 0.017. The negative coefficients indicate that the relative placement advantage weakened as attitudes toward conventional advertising became more favorable (Qayyum et al., 2023).

#### 3.3.1 Conditional effects at low AttADV

At one standard deviation below the AttADV mean, placement produced more favorable brand attitudes than 30-s advertising for both brands. The conditional effect was *b* = 0.17, *t* = 2.68, *p* < 0.01 for Heinz and *b* = 0.27, *t* = 2.15, *p* < 0.05 for Snapple. Thus, the clearest placement advantage occurred among viewers who were comparatively unreceptive to conventional advertising.

#### 3.3.2 Conditional effects at mean AttADV

At the AttADV mean, the placement-versus-advertising contrast did not reach statistical significance for Heinz (*b* = 0.21, *t* = 1.56, *p* > 0.05) or Snapple (*b* = 0.07, *t* = 0.85, *p* > 0.05). To evaluate whether this non-significant result reflected genuine negligible difference or limited statistical sensitivity, TOST equivalence tests were conducted using a pre-specified equivalence bound of *d* = ±0.20. The equivalence tests produced mixed results across the two brands. For Heinz, the lower-bound test was significant but the upper-bound test was not, *t*(143) = 3.24, *p* < 0.001, and *t*(143) = −0.12, *p* = 0.453, respectively, leaving the equivalence test inconclusive. For Snapple, both one-sided tests were significant, *t*(149) = 4.12, *p* < 0.001, and *t*(149) = −2.45, *p* = 0.008, supporting equivalence within the *d* = ±0.20 bound. Thus, the data at mean AttADV provide evidence of practically negligible format differences for Snapple but remain inconclusive for Heinz.

#### 3.3.3 Conditional effects at high AttADV

At one standard deviation above the AttADV mean, neither brand showed a statistically significant placement advantage: *b* = −0.03, *t* = −0.32, *p* > 0.05 for Heinz and *b* = −0.11, *t* = −0.90, *p* > 0.05 for Snapple. The numerically negative coefficients do not support a conclusion that conventional advertising became superior at high AttADV (Sen et al., 2025); the absence of a statistically significant result should not be treated as evidence of format equivalence without direct equivalence testing. TOST equivalence tests using a bound of *d* = ±0.20 were therefore conducted for these high-AttADV conditional effects. The tests confirmed equivalence within *d* = ±0.20 for Heinz, *t*(143) = 2.09, *p* = 0.019, and *t*(143) = −2.73, *p* = 0.004, indicating that the format difference at high advertising receptivity was negligible in practical terms. In contrast, equivalence was not confirmed for Snapple, with *t*(149) = 1.33, *p* = 0.094, and *t*(149) = −3.13, *p* = 0.001, indicating that the data at high AttADV were inconclusive rather than supportive of a negligible format difference. Table 3 reports the conditional effects, and Figure 2 visualizes the change in the placement-versus-advertising contrast across AttADV levels. When attitude toward brand placement (AttBP) was included as a covariate, the exposure format × AttADV interaction remained statistically significant for Heinz, *b* = −0.15, SE = 0.067, *t*(142) = −2.24, *p* = 0.027, Δ*F*(1,142) = 5.02, *p* = 0.027, and for Snapple, *b* = −0.16, SE = 0.067, *t*(148) = −2.39, *p* = 0.018, Δ*F*(1,148) = 5.71, *p* = 0.018. This indicates that AttADV moderated format effects independently of participants’ general favorability toward brand placement, supporting the discriminant contribution of AttADV as a boundary condition.

**Table 3:** Conditional effects of exposure format on brand attitude across levels of AttADV.

AttADV level	Heinz *b* (*t*)	Snapple *b* (*t*)	TOST assessment	Interpretation
Low (M − 1 SD)	0.17 (2.68**)	0.27 (2.15*)	—	Placement significantly more favorable
Mean	0.21 (1.56)	0.07 (0.85)	Heinz: Inconclusive; Snapple: Equivalent	No significant difference; equivalence supported for Snapple
High (M + 1 SD)	−0.03 (−0.32)	−0.11 (−0.90)	Heinz: Equivalent; Snapple: Inconclusive	No significant difference; equivalence supported for Heinz

**Figure 2:**
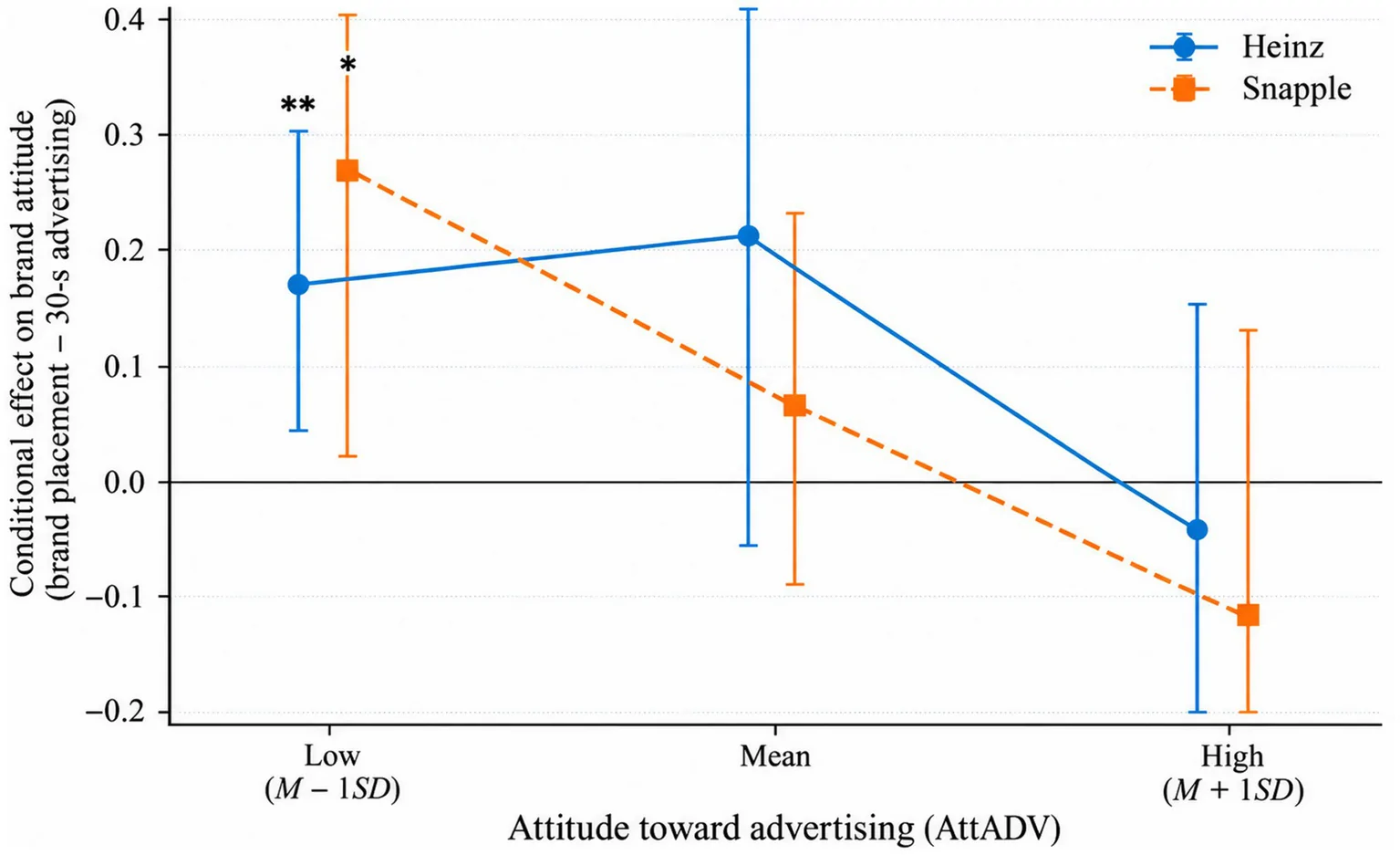
Conditional effect of brand placement versus 30-s advertising on brand attitude across levels of AttADV. Positive values indicate a placement advantage. The placement advantage is statistically significant at low AttADV for both Heinz and Snapple and is not significant at mean or high AttADV. *p < 0.05; **p < 0.01.

### 3.4 Brand attitudes across exposure conditions

For Heinz, mean brand attitude was highest in the placement condition (M = 6.22, SD = 0.80), followed by advertising (M = 5.84, SD = 1.15), combined exposure (M = 5.69, SD = 1.08), and control (M = 5.18, SD = 1.25). Snapple showed the same placement-first descriptive pattern: placement (M = 5.43, SD = 1.04), combined exposure (M = 5.22, SD = 1.46), advertising (M = 5.12, SD = 1.37), and control (M = 4.74, SD = 1.15). The condition means are reported in Table 1 and displayed in Figure 3.

**Figure 3:**
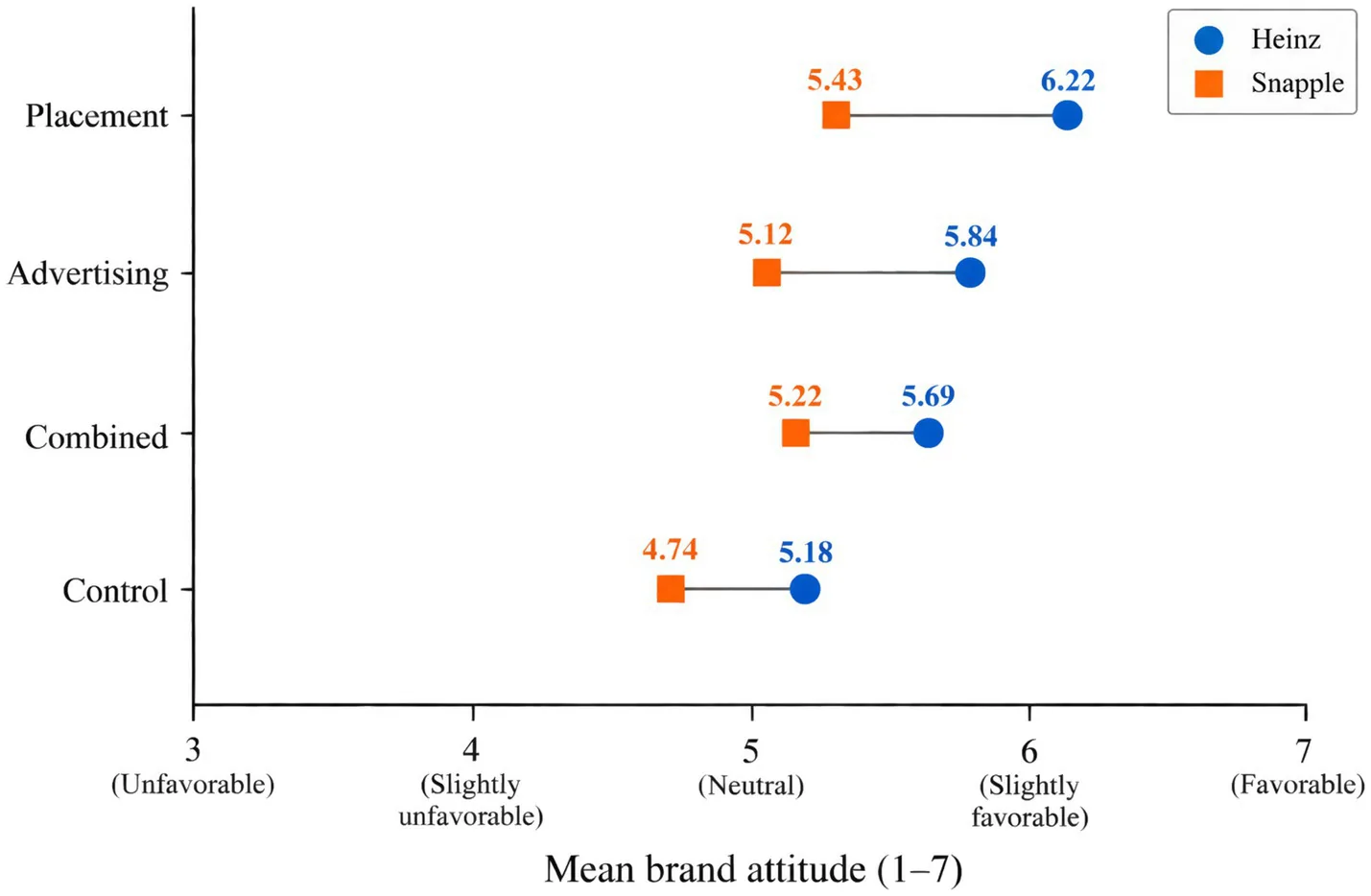
Mean brand attitude by exposure condition for Heinz and Snapple. Values are descriptive means on the seven-point brand-attitude measure; inferential comparisons among the three active conditions.

### 3.5 Purchase intentions across exposure conditions

For Heinz, purchase intention was highest following placement (M = 5.80, SD = 1.19), followed by advertising (M = 5.72, SD = 1.27), combined exposure (M = 5.69, SD = 1.44), and control (M = 4.85, SD = 1.72) (Yannopoulou et al., 2024). For Snapple, placement and advertising produced the same mean purchase intention (both M = 4.74; SD = 1.67 and 1.78, respectively). Combined exposure produced a lower mean (M = 4.43, SD = 1.81), and control produced the lowest mean (M = 3.77, SD = 1.85). Figure 4 presents these descriptive differences.

**Figure 4:**
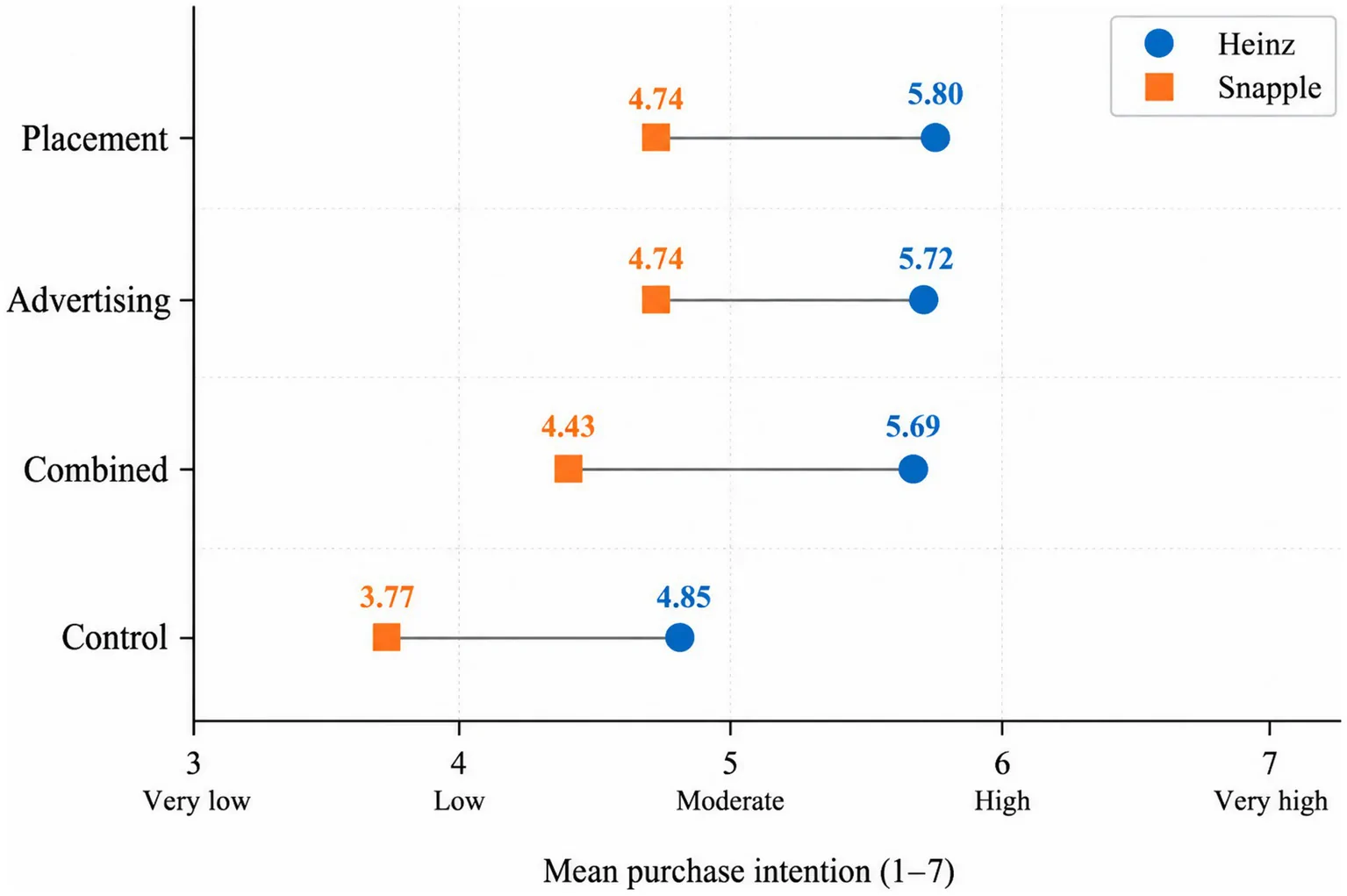
Mean purchase intention by exposure condition for Heinz and Snapple. Values are descriptive means on the seven-point purchase-intention measure; inferential comparisons.

### 3.6 Incremental effect of combined exposure

The three active exposure conditions did not differ significantly in brand attitude for Heinz, *F*(2,233) = 1.95, *p* = 0.144, η^2^ = 0.016, or Snapple, *F*(2,246) = 0.54, *p* = 0.583, η^2^ = 0.004. The same pattern held for purchase intention: the omnibus comparison was non-significant for Heinz, *F*(2,223) = 1.04, *p* = 0.355, η^2^ = 0.009, and Snapple, *F*(2,236) = 0.49, *p* = 0.613, η^2^ = 0.004. Post-hoc power estimates indicated that the present sample was adequately sensitive to medium effects (*f* = 0.25; estimated power > 0.80), but had limited sensitivity to small effects (*f* = 0.10), with estimated power of approximately 0.26 for Heinz brand attitude and 0.27 for Snapple brand attitude. No statistically detectable incremental advantage of combined exposure over single-format exposure was found in the current sample (Rai et al., 2025). Notably, the descriptive means for both brands placed the combined condition numerically below the placement-only condition a pattern consistent with redundancy or dilution of placement-based processing although the statistical tests did not support a definitive interpretation and the observed effect sizes were small. Accordingly, the null omnibus results should be interpreted as evidence against a medium-sized incremental effect, rather than as definitive evidence that combined exposure produces no incremental benefit of any magnitude. Full cell means, standard deviations, sample sizes, and effect size estimates are reported in Table 1.

## 4 Discussion

This study examined how embedded and interruptive brand communication shape audience evaluations and purchase-related intentions, with AttADV treated as an individual-difference boundary condition (Kumar et al., 2025). Three findings organize the discussion. First, participants expressed more favorable general attitudes toward brand placement than toward conventional television advertising. Second, placement outperformed a 30-s advertisement on brand attitude primarily among viewers with relatively unfavorable AttADV; at mean and high AttADV, the difference was not statistically reliable (Chang et al., 2026; Jin and Tao, 2025). Third, combining a placement with a conventional advertisement did not significantly improve brand attitude or purchase intention beyond single-format exposure. Together, the results indicate that persuasive effectiveness is better understood as an audience-by-format relationship than as a fixed property of a particular advertising format (Chen et al., 2025; Ford et al., 2023).

### 4.1 Embedded versus interruptive persuasion

The more favorable general evaluation of brand placement is consistent with the psychological distinction between commercial content that is integrated into a chosen media experience and advertising that interrupts it (Peng et al., 2024). A conventional commercial clearly marks a shift from program content to persuasive communication, whereas a placement is processed within the same narrative stream as characters and events. Earlier brand placement research demonstrated that plot connection and presentation modality can shape brand memory and attitude, while integrative frameworks have emphasized the combined influence of executional and audience factors. The present findings add to this literature by showing that the audience’s general orientation toward advertising helps determine when the format distinction matters (Bashar et al., 2026).

The full-program design is relevant to this interpretation. Media-context research shows that emotional and cognitive reactions to television content can spill over into subsequent advertising responses. Similarly, embedded digital formats can attract different attitudes and attention patterns than conventional display advertising (Li et al., 2026). Although the current experiment examined television rather than digital media, the findings are consistent with the broader proposition that persuasive messages may be evaluated differently depending on the media context in which audiences encounter them.

### 4.2 AttADV as a boundary condition

The most consequential result is the moderation by AttADV. Placement produced more favorable brand attitudes than advertising among participants low in AttADV for both Heinz and Snapple, but this advantage disappeared at average and high levels (Panteli et al., 2024). The finding therefore qualifies any simple claim that placement is inherently more persuasive. Instead, embedded exposure appears to be especially useful when the audience begins from a relatively unfavorable orientation toward conventional advertising. This pattern is consistent with predictions from the Persuasion Knowledge Model, though the mechanism was not directly tested in the present study. One theoretical account is that explicit commercials make the advertiser’s persuasive purpose highly salient, and viewers who already dislike advertising may be more inclined to discount the message or disengage though this process was not directly measured in the current study. Under this interpretation, a brand encountered within entertainment content may attract less format-based resistance because attention remains directed toward the program rather than toward a recognized persuasive attempt a theoretical possibility that future studies should test with direct measures of persuasion knowledge activation. These processes persuasion knowledge activation, counterarguing, and reactance were not measured and should be treated as candidate mechanisms rather than demonstrated mediators.

Research on native advertising provides a parallel observation: once users notice even small cues that identify content as advertising, engagement can decline rapidly. The empirical conclusion is narrower: AttADV systematically changes the placement-versus-advertising relationship for brand attitude. This is also consistent with evidence that general advertising attitudes influence advertising effectiveness and with broader work linking advertising beliefs to consumers’ general evaluations of the institution of advertising.

The non-significant contrasts at mean and high AttADV are equally informative. When viewers are relatively accepting of advertising, an explicit commercial does not carry the same attitudinal disadvantage, leaving less room for placement to outperform it. The small negative conditional coefficients at high AttADV should not be interpreted as evidence that advertising becomes superior, because those differences were not statistically significant. The more defensible conclusion is one of convergence: the observed placement advantage diminished as advertising receptivity increased in this sample, though replication is needed before generalizing this pattern.

### 4.3 Focal brand attitudes and consumer decision-related outcomes

The descriptive condition means show that placement produced the highest brand-attitude score for both focal brands and the highest purchase-intention score for Heinz. Snapple produced identical purchase-intention means in the placement and advertising conditions. These patterns reinforce the value of distinguishing brand attitude from purchase intention. A favorable evaluation is an important proximal response to persuasion, but purchase intention incorporates additional considerations such as need, price, product availability, familiarity, and category involvement. The strongest moderation evidence in this study applies specifically to brand attitude. Purchase intention was examined through condition comparisons rather than an equivalent AttADV moderation model. The results should therefore not be generalized to claim that AttADV moderates purchase intention in the same way. Future studies can test whether the audience-by-format interaction extends from evaluation to stated intention and, ultimately, to observed purchase behavior.

### 4.4 Why combined exposure did not add value

A second contribution is the absence of an incremental benefit from combined exposure. Across both brands, adding a conventional advertisement to placement did not significantly improve brand attitude or purchase intention. This finding is inconsistent with a simple cumulative-exposure assumption in which more brand contacts automatically produce stronger outcomes. Several explanations are plausible. Repetition across formats may have been redundant: once the focal brand had been processed within the program, the subsequent advertisement may have contributed little new information or affective value. A second possibility is that an explicit advertisement following a placement makes the commercial coordination of the exposure more apparent, which could increase recognition of persuasive intent—though neither perceived coordination nor persuasion recognition was measured and these accounts remain speculative.

Contextual advertising research similarly suggests that favorable responses depend partly on how advertising fits the user’s ongoing media experience. These explanations remain speculative because the study did not directly measure perceived redundancy or persuasion recognition. Importantly, the non-significant omnibus tests do not establish that combined exposure was harmful. Several combined-exposure means were descriptively below the placement-only means, but those differences were not statistically reliable. The appropriate conclusion is therefore that the study found no incremental advantage from using both formats within the same program.

### 4.5 Theoretical implications for media and consumer psychology

The study contributes to media and consumer psychology in three ways. First, it reframes the comparison between placement and conventional advertising as an interaction problem. Asking which format is better on average is less informative than asking which format performs better for a particular audience. The observed moderation across both Heinz and Snapple is consistent with AttADV functioning as a meaningful boundary condition for the placement advantage, though the generalizability of this pattern requires testing across broader brand and media contexts. Second, the results connect media context with a broad audience disposition, though the psychological processes linking AttADV to differential format responses remain to be tested directly. AttADV is not a reaction to the focal message; it reflects a pre-existing orientation toward advertising as a communication practice. The findings therefore show that persuasion unfolds within both a media context and a psychological context created by prior beliefs and evaluations.

This perspective complements work on contextual and native advertising that emphasizes the fit between persuasive content and the surrounding environment. Third, the consistency of the moderation pattern across two familiar consumer brands provides evidence that the interaction was not confined to one specific brand within the experiment. This cross-brand consistency should still be interpreted cautiously because both brands are established, relatively low-involvement products. The result provides a basis for broader testing rather than evidence of universal generalizability.

### 4.6 Practical implications

For advertising strategy, the findings imply that audience receptivity should be considered alongside media format. Brand placement may be particularly useful when the intended audience is relatively skeptical of or resistant to conventional advertising. Among advertising-receptive audiences, the difference between placement and a conventional commercial may be comparatively small, so format selection can be guided by other considerations such as cost, reach, narrative fit, and campaign objectives. The combined-exposure result also cautions against assuming that additional exposure necessarily creates synergy. In this experiment, using both a placement and a conventional advertisement did not outperform single-format exposure. The study does not include cost or return-on-investment data, so it cannot determine the economic efficiency of the alternatives. It does indicate that media planners should evaluate the incremental contribution of each additional exposure rather than treat repetition across formats as automatically beneficial.

### 4.7 Limitations and future research

Several limitations constrain interpretation. Of these, one concerns sample attrition: formal attrition analyses were not feasible because demographic data were not collected from the 248 participants who did not complete the survey, leaving open the possibility of systematic differences between completers and non-completers that could not be assessed. First, the experiment used two familiar food and beverage brands within one sitcom. Effects may differ for unfamiliar brands, services, luxury products, high-involvement purchases, or categories associated with greater perceived risk. Additionally, the stimulus program Two and a Half Men has been off the air since 2015, and younger participants in the sample may have had limited familiarity with or affective engagement with the content; reduced narrative engagement could have attenuated placement effects relative to a currently airing program. The two focal placements also differed in character-product interaction: in the Snapple condition, the principal character visibly consumed the product, whereas in the Heinz condition, the character handled but did not consume it. Both brands are food and beverage products and could plausibly have been depicted identically; this asymmetry introduces a confound that limits direct comparison of the two brands’ placement effects and should be corrected in future stimulus development. The high baseline brand attitudes observed for both focal brands Heinz (M = 5.85) and Snapple (M = 5.12) on a seven-point scale raise the possibility that ceiling artifacts contributed to the null combined-exposure finding, as limited scale headroom may have constrained the detection of incremental effects. Future work should test whether AttADV operates similarly across levels of brand familiarity and product involvement. Second, the use of full-length television content improves contextual realism relative to isolated clips, but the viewing experience remained experimental. Everyday media use includes multitasking, second-screen activity, skipping, scrolling, and self-selected content. Extending the design to streaming platforms, social-media feeds, influencer content, and short-form video would test whether the same audience-by-format mechanism operates in contemporary hybrid environments. Recent work has shown that narrative framing features including first- versus third-person perspectives and visual-temporal matching interact with contextual factors to drive narrative transportation and processing fluency in digital brand communication, suggesting that future studies should examine how such features moderate the AttADV-by-format interaction in non-television contexts (Huang et al., 2026). The serial position of the focal advertisement within the commercial break was not systematically varied or reported, and prior research indicates that ad position can influence attention and recall. Future studies should control and report this factor explicitly. A further limitation concerns the absence of direct process measures. The theoretical account offered in this paper draws on the Persuasion Knowledge Model and attributes the observed moderation to differential activation of persuasion knowledge, counterarguing, and perceived intrusiveness. None of these processes were measured. AttADV functions here as a boundary condition — an individual difference that predicts when format differences emerge rather than as evidence of the underlying mechanism. Future studies should include validated measures of persuasion knowledge activation, perceived persuasive intent, ad intrusiveness, and reactance, and should test whether these variables mediate the interaction between AttADV and exposure format.

Third, the overlap between attitude toward conventional advertising (AttADV) and attitude toward brand placement (AttBP) was not fully disentangled in the primary moderation analysis. When AttBP was included as a covariate in supplementary models, the exposure format × AttADV interaction held with comparable magnitude for both Heinz, *b* = −0.15, SE = 0.067, *t*(142) = −2.24, *p* = 0.027, and Snapple, *b* = −0.16, SE = 0.067, *t*(148) = −2.39, *p* = 0.018, indicating that AttADV contributed to the moderation independently of general placement favorability. Future studies should develop measures that more cleanly separate receptivity to embedded formats from receptivity to interruptive advertising and should include both constructs in moderation models from the outset. Fourth, brand attitude and purchase intention were measured shortly after exposure. The design therefore does not establish the durability of the effects or their relationship to actual purchasing. Longitudinal studies could examine delayed brand evaluations, information search, product choice, and observed behavior.

Fifth, moderation was tested for brand attitude but not equivalently for purchase intention. Subsequent studies should determine whether AttADV is primarily an evaluative boundary condition or whether it also alters behavioral intentions. Finally, placement prominence, plot connection, modality, repetition, character-brand congruence, disclosure, narrative engagement, and prior brand experience may interact with AttADV and should be incorporated into more differentiated models.

## 5 Conclusion

Audience responses to brand communication were jointly shaped by exposure format and pre-existing advertising receptivity. In this experiment, brand placement was associated with more favorable brand attitudes than conventional television advertising overall, but its clearest advantage occurred among viewers with relatively unfavorable AttADV. At mean and high AttADV, the placement-versus-advertising difference was not statistically significant. In addition, combining placement with a conventional advertisement did not produce a significant incremental benefit for brand attitude or purchase intention. The findings therefore support a conditional account of persuasive effectiveness. The results suggest that embedded communication may be most advantageous when explicit advertising encounters greater audience resistance, whereas format differences appear to narrow among viewers who are more receptive to advertising a pattern that warrants replication across broader media contexts and brand categories. By identifying AttADV as a boundary condition and demonstrating the pattern across two brands in a full-program setting, the study provides a focused framework for future research on how audience dispositions and media context jointly shape consumer evaluations and decision-related responses.

## Data Availability

The raw data supporting the conclusions of this article will be made available by the authors, without undue reservation.
